# Tumor-Associated Macrophages: Key Players in Triple-Negative Breast Cancer

**DOI:** 10.3389/fonc.2022.772615

**Published:** 2022-02-14

**Authors:** Xia Qiu, Tianjiao Zhao, Ran Luo, Ran Qiu, Zhaoming Li

**Affiliations:** ^1^ Department of Oncology, The First Affiliated Hospital of Zhengzhou University, Zhengzhou, China; ^2^ Department of Cell Biology, Wuhan Institute of Bioengineering, Wuhan, China

**Keywords:** TNBC, TAMs, ER, PR, HER-2

## Abstract

Triple negative breast cancer (TNBC) refers to the subtype of breast cancer which is negative for ER, PR, and HER-2 receptors. Tumor-associated macrophages (TAMs) refer to the leukocyte infiltrating tumor, derived from circulating blood mononuclear cells and differentiating into macrophages after exuding tissues. TAMs are divided into typical activated M1 subtype and alternately activated M2 subtype, which have different expressions of receptors, cytokines and chemokines. M1 is characterized by expressing a large amount of inducible nitric oxide synthase and TNF-α, and exert anti-tumor activity by promoting pro-inflammatory and immune responses. M2 usually expresses Arginase 1 and high levels of cytokines, growth factors and proteases to support their carcinogenic function. Recent studies demonstrate that TAMs participate in the process of TNBC from occurrence to metastasis, and might serve as potential biomarkers for prognosis prediction.

## Introduction

Triple negative breast cancer (TNBC) refers to the subtype of breast cancer which is negative for ER, PR, and HER-2 receptors, accounting for approximately 12%-17% of invasive breast cancer (1). Compared with other breast cancer subtypes, TNBC has the highest recurrence rate and metastasis rate. Chemotherapy is most common treatment for TNBC currently while drug resistance, non-target characteristics and severe systemic side effects lead to ineffective prognosis for patients with metastatic cases (2, 3). Therefore, it is necessary to discover cutting-edge and effective treatment strategies for TNBC.

In recent years, the treatment strategy based on the interference and remodeling of the tumor microenvironment (TME) has gradually emerged (4). With the deepening of research on TME, it is found that immune cells in TME play a complex and non-negligible role in tumor progression. Among them, tumor-associated macrophages (TAMs) are such a kind of important components (5, 6). TAMs participate in the process of TNBC from occurrence to metastasis, and have potential value in evaluating disease-free survival and overall survival of TNBC (7, 8).

**Figure 1:**
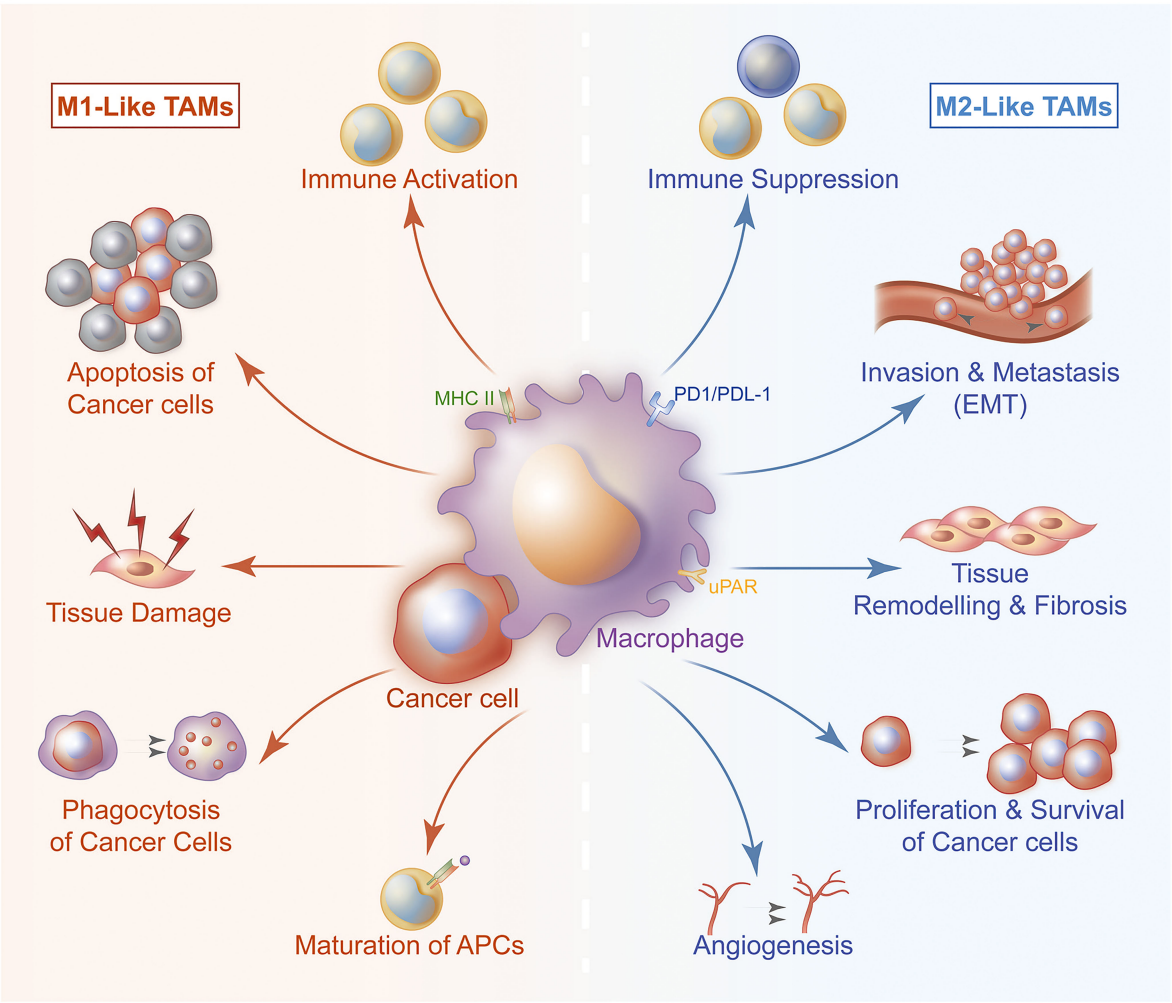
TAMs are divided into typical activated M1 subtype and alternately activated M2 subtype. The two subtypes participate in the process of TNBC from occurrence to metastasis of TNBC. M1 exerts anti-tumor activity by promoting pro-inflammatory and immune responses. M1 can present antigen to immune cell, then these cells are activated and directed to kill tumor cells. M1 can also secrete cytokines to induce tumor cells apoptosis. M2 is involved in stimulating tumor angiogenesis, matrix remodeling, tumor cell migration and invasion, it also plays a significant role in immune suppression and tissue repair.

TAMs refer to the leukocyte infiltrating tumor, derived from circulating blood mononuclear cells and differentiating into macrophages after exuding tissues (9–12). Increasing evidence indicates that macrophages are not homogeneous. They can be divided into specific subgroups based on polarization requirements, phenotype and function. TAMs are divided into typical activated M1 subtype and alternately activated M2 subtype, which have different expressions of receptors, cytokines and chemokines (13–15). M1 is characterized by expressing a large amount of inducible nitric oxide synthase and TNF-α, and exert anti-tumor activity by promoting pro-inflammatory and immune responses (5, 16, 17). M2 usually expresses Arginase 1 and high levels of cytokines, growth factors and proteases to support their carcinogenic function (18, 19). In addition, M2 is involved in stimulating tumor angiogenesis, matrix remodeling, tumor cell migration and invasion, and promoting immune suppression (7, 8) (**Figure 1**).

## Polarization and Induction of TAMs

Circulating precursors of macrophages derived from bone marrow are recruited into the tumor microenvironment which are affected by inflammatory mediators and chemokines to participate in the tumor immune response (20, 21). Recruited TAMs can differentiate into macrophages with different phenotypes and functions under diverse activation conditions. Stimulation of Lipopolysaccharide (LPS), IFN-γ, etc. is the classical pathway to activate macrophages (M1 type); Stimulation of anti-inflammatory factors such as IL-10 and TGF-β, is a non-classical pathway to activate macrophages (M2) (**Table 1**) (21–25).

**Table 1:** Polarization of Macrophage and its function.

Phenotypes	Stimuli	Markers	Functions	References
M1	IFN-γ, LPS, GM-CSF, TNF-α	CXCL9, IL-12, IL-6, IL-23, iNOS, CD80, CD86, TNF-α	Pro-inflammation, microbicidal effect, tumor resistance	(21–28)
M2	IL-4, IL-13, IL-6, IL-10, Glucocorticoids, immunoglobulin complexes	CD163, CD204, CD206, CCL17, CXCL13, IL-1R, VEGF, IL-10, TGF-α	Anti-inflammatory, wound healing, angiogenesis, immunosuppression, tumor progression and invasion	(21–25, 29–33)

IFN, interferon; LPS, lipopolysaccharide; GM-CSF, granulocyte-macrophage colony stimulating factor; TNF, tumor necrosis factor; CXCL, chemokine (CX-C motif) ligand; iNOS, inducible nitric oxide synthase; IL, interleukin; CD, cluster of differentiation; CCL, chemokine (C-C motif) ligand; VEGF, vascular endothelial growth factor.

M1 macrophages are highly effective pro-inflammatory immune effector cells that release superoxide anions and nitrogen free radicals after injury or inflammatory activation (26, 27). It plays a role of extracellular killing and present antigens to T cells triggering anti-tumor effects. IL-1β is a cytokine, with the concentration-dependent anti-tumor effect, secreted by macrophage. LPS can activate macrophages to secrete IL-1β. Cytolysin A (ClyA), secreted by Gram-negative bacteria, have been proven to induce IL-1β secretion, which can enhance the tumoricidal activities (28). M2 macrophages have a negative regulatory effect on tumor immunity by repairing damaged tissues and inhibiting inflammation (29). In the tumor environment, IL-10 and TGF-β can transform macrophages from M1 phenotype into M2 phenotype (15–17, 26, 34) (**Figure 1**). Not only the specific cytokines or factors can render the macrophage become M2, injury or damage can also make macrophage polarize to M2. In return, M2 secrete Arginase 1 (Arg1), VEGF and TNF-α to repair damage through the CXCL-10/CXCR3 pathway (30).

Accumulated evidence have shown that TAMs in the tumor tissues tend to polarize to M2 type once they affected or interact with tumor extracellular matrix (23, 34). By co-inoculating macrophages RAW264.7 and triple-negative breast cancer cells 4T1 into the mammary ducts of mice, process of TNBC progression from carcinoma *in situ* to invasive carcinoma was simulated (35–37). It was found that the expression level of IL-12 related to M1 macrophages in the co-inoculation group was significantly lower than that in the macrophage alone group during the process of cell inoculation to tumor cells and breaking through the duct basement membrane (38, 39). The level of TGF-β1, a M2- related cytokine, was significantly increased, accompanied by distant lymph node and lung metastasis. In addition, an increasing level of MMP-8 and VEGF in the peripheral blood of mice was also observed. MMP-8 and VEGF are important M1/M2 polarization inducing factors (37). Therefore, under the induction of tumor cells, M2 polarization of TAMs can be considered as an alternating positive feedback process (**Figure 1**).

## Tumor Immunosuppression and Immune Escape

Macrophages have strong phagocytic ability and antigen presentation ability which play an important role in connecting innate immunity and adaptive immunity (40–45). In TME, TAMs switch from M1 subtype with tumor killing function to M2 subtype with tissue repair function, which greatly weakens tumor killing ability of tumor system (18, 44, 46–49). Transformation from M1 subtype to M2 subtype can limit inherent recognition and phagocytic abilities of macrophages and tumor-killing ability of CD4+ T cells and CD8+ T cells that cooperate with them. It can also activates Treg cells and helper T cells causing tumor immunosuppression (50–58) (**Figure 1**).

As a regulatory factor which limits killing effect of T cells, PD-1 is widely present in a variety of T cells (59–63). PD-L1 is the receptor of PD-1, which is mostly expressed on the surface of tumor cells and macrophages (61, 64–66). TNBC cells can highly express PD-L1 so that their T cell killing effect in the tumor environment is significantly inhibited (56, 67–70). TAMs can secrete a variety of cytokines in the TNBC environment, which mediate their immunosuppressive and tumor-promoting activities (71–73). Studies have found that TAMs can secrete IFN-γ through JAK/STAT3 and PI3K/AKT signaling pathways, thereby inducing the expression of PD-L1 (74, 75). IL-6 is related to growth of TNBC and prognosis of patients. In the absence of IL-6, expression of PD-L1 is enhanced and the anti-PD-L1 antibody’s inhibitory activity *in vivo* is more significant (76–79). IL-18 is a pleiotropic cytokine member of the IL-1 family which has pro-inflammatory and anti-inflammatory functions (77, 80). It is produced by a variety of cell types including macrophages. Tumor-derived IL-18 levels are significantly related to the low survival rate of TNBC patients (81–83). TGF-β is a multifunctional cytokine, which participates in the production of Treg in the mouse tumor microenvironment and supports its suppression of effector T cells (18). TGF-β can also increase its inhibitory activity by inducing polarization of TAM to M2 phenotype, and induce the up-regulation of PD-L1 leading to tumor escape (84, 85). In addition, TAMs can also promote the development and activity of PD-1+Treg, and then participate in TNBC tumor immune escape (55, 86). PD-1 was also reported to be expressed on the surface of TAMs and mainly exist as type M2 (56, 87–89). Compare to CD68, a surface marker of M2, CD163 and CD260 are major markers with more specificity to help us identify M2 (**Table 1**) (90, 91).

TAMs with high expression of PD-1 have reduced phagocytic ability, which reduce anti-tumor immune effect to a certain extent (89). It blocks PD -1/PD-L1 binding and enhances the phagocytic ability of macrophages to inhibit tumor growth and effectively prolong the survival time of tumor-bearing mice (47). Under induction of tumor cells, TAMs become important mediators and regulatory factors for tumor immunosuppression and immune escape.

## Promoting Tumor Blood Vessel and Lymphatic Vessel Formation

Tumor angiogenesis is an essential part of TNBC proliferation and metastasis (14, 92, 93). TAMs plays an indispensable role in promoting tumor angiogenesis (93). Hypoxia is a typical characteristic of solid tumors (94). Expression of HIF-1 is up-regulated in TNBC, which activates the HIF-CSF pathway and recruits a large number of macrophages to the tumor area (95, 96). This process is a key step in the recruitment of macrophages in TME. Recruited macrophages can participate in various stages of tumor angiogenesis. For example, matrix metalloproteinases and proteolytic enzymes produced by macrophages can reconstruct the extracellular matrix and provide favorable conditions for the formation of new blood vessels (93). Cytokines secreted by macrophages can provide a connecting framework for new blood vessels (20). Macrophages in TME can promote growth of lymphatic endothelial cells and provide support for tumor lymphatic metastasis. Induced by tumor cells, macrophages overexpress β4 integrin which forces macrophages to aggregate and adhere to the proximal end of lymphatic vessels. At the same time, their own expression of TGF-β1 drives the contraction of lymphatic endothelial cells (97) (**Figure 1**). Aggregated macrophages undergo lymphatic remodeling by increasing permeability and destroying surrounding tissues to achieve tumor cell metastasis *via* lymphatic pathways. Macrophages’ tissue function including renewal and remodeling of blood vessels together with lymphatic vasculature, though it improves the aggressiveness of tumors in the tumor environment.

## TAMs and TNBC Migration and Invasion

It has been found that TAMs can enhance tumor cell stemness and increase tumor cell invasiveness by participating in epithelial-mesenchymal transition (EMT) (7, 98–100). Matrix metalloproteinases (MMPs), cysteine cathepsin and serine proteases secreted by TAMs can hydrolyze the extracellular matrix, which is conducive to invasion of tumor cells to surroundings (**Figure 1**). After co-incubating macrophages with different phenotypes with breast cancer cells, it was found that those co-incubated with M1 macrophages appeared as cobblestone-like epithelial-like cells under microscope. Compared with M2 macrophages, co-incubators appear as slenderer mesenchymal-like cells. In addition, E-cadherin in co-incubated with M2 group was significantly higher in co-incubated with M1 macrophages (7, 98, 100–104). It shows that M1 TAMs have the potential to reverse EMT, which can reduce the invasiveness of tumor cells to a certain extent.

## TAMs and Prognosis Prediction

High density of TAMs in TNBC is associated with poor prognosis and indicates a higher risk of metastasis (105). TAMs immunohistochemical staining of tumor tissues found that patients with higher pathological grades were often accompanied by higher TAMs level. Compared with patients with low TAMs infiltration, the overall survival and disease-free survival of patients with higher group was significantly shortened (39, 51, 106). CD163 and CD204 are relatively specific markers for M2, the breast cancer infiltration of CD163 positive and CD204 positive TAMs tends to have a poor prognosis as these TAMs are associated with fast proliferation, poor differentiation (31–33). In addition, infiltrating TAMs also have a certain impact on the efficacy of chemotherapy. Common TNBC chemotherapeutic drugs can activate TAMs, and activated TAMs can promote the repair of damaged tumor tissues, thereby inducing chemotherapy tolerance (107, 108). Not only that, TAMs can produce a large amount of IL-10, which can inhibit the production of IL-12 by dendritic cells and limit the immune killing of tumors with CD8+ T cells (39). Further research found that compared with the number of TAMs, the phenotype of TAMs is more suitable for predicting efficacy of TNBC anthracycline chemotherapeutics (109).

## TAMs-Based Targeted Therapy

In recent years, the targeted therapy of TAMs mainly focuses on inhibiting TAMs recruitment, TAMs depletion, and reversing the polarization of TAMs (110). Blocking the effect of chemokines is an important method to inhibit the recruitment of TAMs, and the current research targets are mostly CCL2/CCR2 (52, 111). The inhibition of the CCL2/CCR2 axis can reduce the mobilization of bone marrow mononuclear cells, thereby reducing the infiltration of macrophages in the breast (112, 113). Studies have shown that trabectedine and bortezomib can inhibit the recruitment of macrophages by reducing the content of CCL2 in plasma (114). CCL5 can induce the recurrence of breast cancer by recruiting macrophages in residual tumors. CCL5 may become an important target for adjuvant chemotherapy and curbing recurrence of TNBC (39, 115, 116). Cytokines can effectively regulate the polarization direction of TAMs. For instance, when TAMs are exposed to cytokines secreted by CD4+Th1 cells (such as TNF, IL-12, etc.), TAMs tend to be polarized as M1 type. The NF-kB pathway is an important pathway that regulates the transcription of CD4+Th1 cytokines. Activating the NF-kB pathway can promote the polarization of TAMs to M1 type, thereby inhibiting the progress of TNBC (18, 117).

Bisphosphonate-based macrophage apoptosis inducers have been widely used in TAMs depletion (118). Bisphosphonate is easily captured by macrophages through endocytosis. The internalized Bisphosphonate can inhibit the activity of famesyl diphosphate (FPP) synthase and induce macrophage apoptosis by limiting the prenylation of RAS-related proteins. Continuous administration of zoledronic acid in a mouse spontaneous breast cancer model can significantly reduce angiogenesis, reduce the density of TAMs, and improve survival. Many clinical trials have shown that bisphosphonate therapy in post-menopausal women with breast cancer have a significant benefit (119). However, it is not applied to the menopausal women (120).

CSF1 and CCL2 play a key role in the generation of TAM and are related to the growth of TNBC tumors (61, 121, 122). Inhibition of CSF1 *in vivo* can reduce TAM infiltration and tumor growth and progression. Blocking CSF-1 can affect the osteoclast production of cancer cells in the co-culture system (123). Similarly, inhibiting CCL2 can block tumor stem cell renewal and M2 recruitment, thereby inhibiting the progression of TNBC (124, 125). This indicates that inhibiting CSF1 and CCL2 may be an effective strategy to reduce the accumulation of TAM. The transcription factor NF-κB can regulate the expression of tumor-promoting genes (IL-6 and TNF-α). By activating the activity of NF-κB through the IKKβ pathway, TAM can be re-cultured to the M1 phenotype (126). Therefore, converting M2 to the anti-tumor M1 phenotype may be a potentially effective strategy for cancer patients.

In addition, regulating the expression of PD-1/PD-L1 by regulating various cytokines secreted by TAMs is also a potential therapeutic strategy (56). For example, JAK/STAT3 signal is related to PD-L1 overexpression induced by IFN-γ. Inhibition of STAT3 signal by WP1066 can reduce tumor-related endothelial angiogenesis and invasion, thereby reducing the incidence of brain metastasis (56). TGF-β is related to M2 polarization and PD-L1 overexpression (48). Therefore, the combination of TGF-β inhibitors and anti-PD-1/PD-L1 specific antibodies is reasonable in clinical practice, and related clinical trials are also underway.

Application of nanoparticle targeted drug delivery systems to traditional TAMs is currently a hot research topic (109, 127). Nanoparticles can carry drugs, metal materials, and miRNAs, which can work together to interfere with TAMs through a variety of mechanisms of action. Studies have found that dextran-coated iron oxide nanoparticles can generate reactive oxygen species (ROS) through the Fenton reaction mediated by iron oxide, which mediates the repolarization of TAMs to M1 macrophages, thereby inhibiting breast cancer progression (128). Incorporation chemotherapy with macrophage-related treatment can enhance the antitumor effect by recruiting macrophage to TAM and induce M2 polarize to M1 (22, 129).

## Conclusion and Perspectives

TAMs are an important component of the tumor microenvironment and occupy a high proportion of immune cells (45). They participate in whole process of TNBC occurrence, development and metastasis by regulating tumor cell immune evasion, tumor blood vessel and lymphangiogenesis (130). The phenotypic transition of TAMs in TME promotes the tumor immune microenvironment to change from an anti-tumor state to an immunosuppressive state. This dynamic change makes TAMs an important part of regulating tumor behavior and feedback on efficacy evaluation. In view of the important role of TAMs in tumor progression, treatment strategies based on TAMs have emerged. Due to the high heterogeneity of TNBC, targeted therapy for a single TAMs-related pathway often comes to failure. In the future, cooperation macrophage-targeted therapy with conventional chemotherapy, immunotherapy and adjuvant therapy maybe a promising choice for TNBC (**Table 2**), and multimodal targeted therapy based on TAMs may become a research hotspot (131).

**Table 2:** Treatment of triple negative breast cancer.

Chemotherapy	Taxus, gemcitabine, capecitabine, vinorelbine and platinum
immunotherapy	PD1 inhibitor, PD-L1 inhibitor, PARP inhibitor
anti-VEGF	Bevacizumab
macrophage-targeted therapy	PI3K suppressors, interleukin therapy, suppression of hypoxia, inhibition of CCL2/CCR2, activation of NF-κB, CSF1 inhibitor
adjuvant therapy	bisphosphate, nanoparticle delivery therapy

## Author Contributions

RQ, RL, XQ, and ZL contributed to the conception of the study and wrote the manuscript. All authors contributed to the article and approved the submitted version.

## Conflict of Interest

The authors declare that the research was conducted in the absence of any commercial or financial relationships that could be construed as a potential conflict of interest.

## Publisher’s Note

All claims expressed in this article are solely those of the authors and do not necessarily represent those of their affiliated organizations, or those of the publisher, the editors and the reviewers. Any product that may be evaluated in this article, or claim that may be made by its manufacturer, is not guaranteed or endorsed by the publisher.

## Abbreviations

TNBC, Triple negative breast cancer; ER, estrogen receptor; PR, progesterone receptor; HER-2, human epidermal growth factor receptor 2; TME, tumor microenvironment; ClyA, Cytolysin A; LPS, Lipopolysaccharide; Arg1, Arginase I; IFN-γ, Interferon-gamma; TAMs, tumor-associated macrophages; TGF-β, Transforming growth factor beta; VEGF, vascular endothelial growth factor; TNF-α, Tumor necrosis factor alpha; IL, Interleukin; iNOS, Inducible nitric oxide synthase; CXCL-10:C-X-C Motif Chemokine Ligand 10 also known as Interferon γ–induced protein-10 (IP-10); CXCR3, C-X-C Motif Chemokine Receptor 3; CSF1, Colony stimulating factor 1; CCL2, Chemokine ligand 2; CCR2, chemokine receptor 2; FPP, famesyl diphosphate; PD-1, Programmed death-1; PD-L1, Programmed death-1 receptor; ROS, reactive oxygen species; EMT, epithelial-mesenchymal transition; MMPs, Matrix metalloproteinases.
